# Unraveling the Variation Pattern of *Oncomelania hupensis* in the Yangtze River Economic Belt Based on Spatiotemporal Analysis

**DOI:** 10.3390/tropicalmed8020071

**Published:** 2023-01-18

**Authors:** Lu Li, Lijuan Zhang, Yinlong Li, Zhong Hong, Qiang Wang, Wangping Deng, Shizhu Li, Jing Xu

**Affiliations:** National Institute of Parasitic Disease, Chinese Center for Disease Control and Prevention (Chinese Center for Tropical Disease Research), NHC Key Laboratory of Parasite and Vector Biology, WHO Collaborating Center for Tropical Disease, National Center for International Research on Tropical Disease, Shanghai 200025, China

**Keywords:** Yangtze River Economic Belt, *Oncomelania hupensis*, spatiotemporal pattern

## Abstract

The construction of the Yangtze River Economic Belt (YEB) is a great national economic development strategy in China. As the YEB covers most endemic provinces of schistosomiasis japonica featured by low endemicity, this study aimed to investigate the spatiotemporal distribution pattern of *Oncomelania hupensis* (*O. hupensis*), which serves as the only intermediate host of *Schistosoma japonicum* in the YEB. Annual data reflecting the distribution of *O. hupensis* from 2015 to 2021 were collected from the National Institute of Parasitic Disease, Chinese Center for Disease Control and Prevention. Spatial autocorrelation analysis, hotspot analysis and space–time scan analysis were performed to explore the aggregation features and spatiotemporal dynamics of the snail distribution. The distribution of both total snail habitats (during 2015–2021) and emerging snail habitats (in 2016, 2018 and 2020) showed spatial autocorrelation (Z = 15.8~16.1, *p* < 0.05; Z = 2.3~7.5, *p* < 0.05). Hotspot (high-value areas in space) counties were mainly clustered in the alluvial plain of the middle and lower reaches of the YEB. Eight spatial and temporal clusters of snail habitats were scanned and were mainly concentrated in the counties of Anhui, Jiangxi, Hubei, Hunan and Jiangsu provinces along the Yangtze River. The YEB carries a tremendous burden of *O. hupensis*. Surveillance and risk identification based on the snail presence should be strengthened to provide reference for protecting humans and public health security in the YEB.

## 1. Introduction

As the largest developing country in the world, China is devoted to the economic development and has given high priority to the construction of the Yangtze River Economic Belt (YEB) [1]. The YEB involves 3 major regions and covers 1069 counties (cities, districts) in 9 provinces and 2 municipalities [2], with a total area of about 2,052,300 square kilometers, accounting for 21.4% of the national land area. Being an inland river economic belt with the largest scale and widest scope of influence in China, the construction and development of the YEB will inevitably drive the flow and exchange of population and resources, which may have profound impacts on the occurrence, transmission and distribution of diseases, especially infectious diseases [3].

There are six *Schistosoma* spp. hosted in human beings, causing diseases named schistosomiasis [4]. The Global Burden of Disease Study 2016 showed that schistosomiasis could lead to 1.86 million disability-adjusted life years (DALYs) losses [5]. Even so, the actual burden was significantly underestimated [6]. Schistosomiasis japonica is a zoonotic disease currently endemic in China, the Philippines and Indonesia [7]. It is caused by the trematode flukes of *Schistosoma japonicum* (*S. japonicum*), which parasitizes in the mesenteric veins of humans or other mammalians, causing hepatointestinal egg granulomas and fibrosis as the main pathological changes [7]. In China, schistosomiasis japonica was one of the critical public health issues threatening people’s health and hindering social and economic development dramatically [8,9]. In the past, it was endemic in 12 provinces, municipalities and autonomous regions in the south of China, mainly along the Yangtze River. Despite great achievements obtained in China with a significant decrease in morbidity and prevalence of schistosomiasis in human beings and livestock, a potential transmission risk still exists due to the spread distribution of *Oncomelania hupensis*, which serves as the only intermediate host of *S. japonicum* in China, in addition, a large variety of wildlife can transmit schistosomiasis and act as reservoir hosts of *S. japonicum* [9,10]. In the regions covered by the YEB, Chongqing City and Guizhou Province are, historically, non-epidemic areas of schistosomiasis. The other nine provinces or municipalities were heavily epidemic areas of schistosomiasis, with the prevalence of schistosomiasis in humans higher than 20% [7]. According to the national criteria for Schistosomiasis Control and Elimination (GB15976-2015) issued by the Chinese government [11], Shanghai and Zhejiang have reached the standard indicating elimination, and Sichuan and Jiangsu provinces interrupted the transmission of schistosomiasis in 2017 and 2019, respectively, after fighting for several decades against schistosomiasis. However, schistosomiasis is still transmitted in five other provinces including Yunnan, Hubei, Hunan, Jiangxi and Anhui, provinces with large snail habitats and sporadic acute cases.

Being a snail-borne parasitic disease [12], schistosomiasis japonica distributes strictly in consistence with the habitats of *O. hupensis* in China [13]. *O. hupensis* is an amphibious freshwater snail and could survive in various environments, including beaches of rivers or lakes, ditches of irrigation systems, dry land, paddy fields, bottomlands, ponds, etc. Its distribution could be impacted by floodings, construction of water conservancy projects, strengthening interventions or human activities. Previous studies showed that the resurgence of snail habitats was usually detected earlier than the rebound in the morbidity of schistosomiasis in human beings and livestock, particularly in areas with very low endemicity. Thus, the dynamic changes of snail distribution could serve as an early warning indicator of a schistosomiasis rebound [14]. In addition, the construction of the YEB is bound to change the environment and impact the transmission of schistosomiasis. Therefore, identifying potential risk areas or key areas for schistosomiasis control and prevention are pivotal to allocate limited resources to reasonably protect human health and ensure public health security in the YEB [15]. In this study, we analyzed the spatiotemporal distribution of *O. hupensis* in the YEB from 2015 to 2021 using spatial autocorrelation analysis and scan analysis to provide reference for formulating reasonable control strategies and allocating health resources.

## 2. Materials and Methods

### 2.1. Study Area

**Figure 1:**
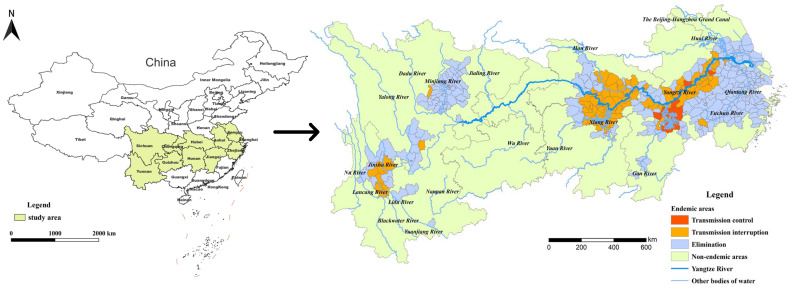
Location of the study area and stage of schistosomiasis control in endemic counties in 2021.

The YEB covers 401 schistosomiasis-endemic counties or county-level districts distributed in Shanghai, Zhejiang, Jiangsu, Anhui, Hubei, Hunan, Jiangxi, Sichuan and Yunnan provinces or province-level municipalities based on the administrative division in 2021 (Figure 1). By the end of 2021, 289 of the 401 endemic counties (cities, districts) had reached the standard of schistosomiasis elimination, 100 had reached the standard of transmission interruption, while the other 12 counties were still at the stage of transmission control. There were only 3 stools positives from human beings for schistosomiasis in the whole country in 2021, indicating that the prevalence of schistosomiasis in humans had decreased to a very low level.

### 2.2. Data Sources and Processing

The data reflecting the distribution of *O. hupensis* were organized by Microsoft Excel 2019 (Microsoft, Inc., Redmond, WA, USA) and were obtained from the annual database of schistosomiasis prevention and control from 2015 to 2021 constructed by the National Institute of Parasitic Diseases, Chinese Center for Disease Control and Prevention. In China, snail surveys are conducted mainly in spring but, in some places, also in autumn each year. Variables included the name of the endemic areas, the administrative division code and land area, the area of the snail habitats, the area of emerging snail habitats, the area of snail habitats with infected snails, the major ecotype of the snail habitats and the stage of schistosomiasis control according to the criteria of schistosomiasis control and elimination (GB15976-2015) [11] issued by the Ministry of Health of China. After collecting geographic vector data, the complete vector layer of the study areas was registered and sorted out. ArcGIS 10.8 (ESRI, Inc., Redlands, CA, USA) software was used to associate the snail data according to the administrative division code of the endemic counties (cities, districts), and a spatial database of *O. hupensis* was constructed.

### 2.3. Descriptive Analysis

The overall trends of snail burden were obtained by Origin 2022 (OriginLab, Inc., Northampton, MA, USA) software by comparing the area of total snail habitats, the area of emerging snail habitats and the area of snail habitats with infected snails in the YEB.

### 2.4. Global Spatial Autocorrelation Analysis

Global autocorrelation analysis was conducted by ArcGIS 10.8 (ESRI, Inc., Redlands, CA, USA) to investigate whether spatial aggregation existed for the snail distribution in the YEB in general. The weights in a spatial weight matrix were adjusted by a standardization technique. We chose the inverse distance method as the parameter for conceptualizing spatial relationships. The value of the global index Moran’s I ranges between −1 (maximum negative correlation) and 1 (maximum positive correlation), with a value of 0 indicating a random spatial distribution [16,17]. A test statistic Z-score larger than 1.96 or less than −1.96 and a two-side *p*-value less than 0.05 were considered statistically significant.

### 2.5. Hotspot Analysis

The actual location of significant spatial clusters and outliers of the snail habitats in the YEB were detected by local indicators of spatial association (LISA) including Anselin Local Moran’s I and Getis-Ord Gi* [18]. The local Moran’s I, like the global Moran’s I, ranges from −1 to 1, with negative values indicating the existence of unlike values clustering, zero meaning spatial randomness, and positive values meaning the spatial clustering of similar values. Analysis was conducted to classify snail habitats into hotspots (high–high cluster, H-H cluster), coldspots (low–low cluster, L-L cluster), and spatial outlier (high–low or low–high cluster, H-L or L-H cluster) [19,20]. The Gi* statistic is based on a weighted distance matrix, and its statistical significance is judged by Z-score and *p*-value, which were used to determine the intensity and stability of hotspots or coldspots [21]. Locations with a statistically significant and large Z-score tend to have a more intense cluster of high-value areas (hotspots), while those with a small Z-score tend to have a more intense cluster of low-value areas (coldspots) [21,22]. ArcGIS 10.8 (ESRI, Inc., Redlands, CA, USA) was used for all the above spatial analysis.

### 2.6. Spatiotemporal Analysis

A retrospective space–time analysis was performed to identify areas and periods with a significantly higher risk than the average risk of snail distribution using Kulldroff’s space–time scan statistics by SaTScan 10.0 (Kulldorff & Information Management Services, Inc., Bethesda, MD, USA) [23]. For the distribution pattern analysis of the snail habitats, the Poisson distribution model was selected for analyzing the indicator of the proportion of the area of the snail habitats (the area of the snail habitats divided by the area of the administrative unit where the snail habitats were located, rounded to an integer) [24]. The Bernoulli model was used to analyze the emerging snail habitats to discover the spatial and temporal distribution of the snails. According to the data requirements of the Bernoulli model, the value of “1” was assigned to the counties with an area of emerging snail habitats larger than zero, and “0” was assigned to the counties without emerging snail habitats detected in that year. A dynamic cylinder scanning window was established, and the maximum spatial cluster size was set to 20% of the total area at risk and to 50% of the study period, with data aggregated by counties and year, respectively [25]. The log-likelihood ratio (LLR) was tested by the Monte Carlo Method with a test level α = 0.05. When *p* < 0.05, the relative risk (RR) difference inside and outside the scanning window was considered statistically significant. The window with the largest LLR value was considered the most likely cluster, and the remaining clusters were regarded as secondary clusters [26]. The figures presenting the snail distribution were mapped by ArcGIS 10.8 (ESRI, Inc., Redlands, CA, USA).

## 3. Results

### 3.1. General Information

**Figure 2:**
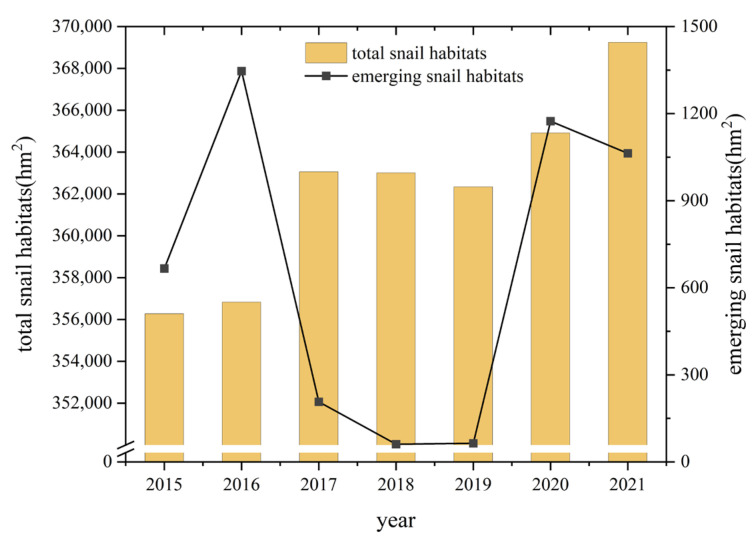
Changes of snail burden in the YEB during 2015–2021.

From 2015 to 2021, the total area of snail habitats distributed in YEB increased from 356,279.57 hm^2^ to 369,244.05 hm^2^, with an increase of 3.65%. Classified by the ecotype of the snail habitats, 94.72% of the snail habitats (349,737.78 hm^2^) were distributed in marshland and lake regions, 5.19% of them (19,156.73 hm^2^) were located in hilly and mountainous regions, while the others were in waterway network regions in 2021 [27]. The area of emerging snail habitats increased from 666.04 hm^2^ to 1063.08 hm^2^ from 2015 to 2021, with high values mainly occurring in 2016, 2020 and 2021 (Figure 2). Infected snails were only found in 2020 in the Guichi District of Anhui Province, in an area of 1.96 hm^2^.

### 3.2. Global Spatial Autocorrelation Analysis

From 2015 to 2021, the global Moran’s I remained stable around 0.49–0.50, with all Z-scores much higher than 1.96 and *p*-values less than 0.05 (Table 1), which indicated a statistically significant positive spatial autocorrelation in snail habitats in the YEB. For the emerging snail habitats, spatial aggregation was only found in 2016, 2018 and 2020, with Moran’s I higher than 0 and Z-score higher than 1.96 (Table 1).

### 3.3. Hotspot Analysis

**Figure 3:**
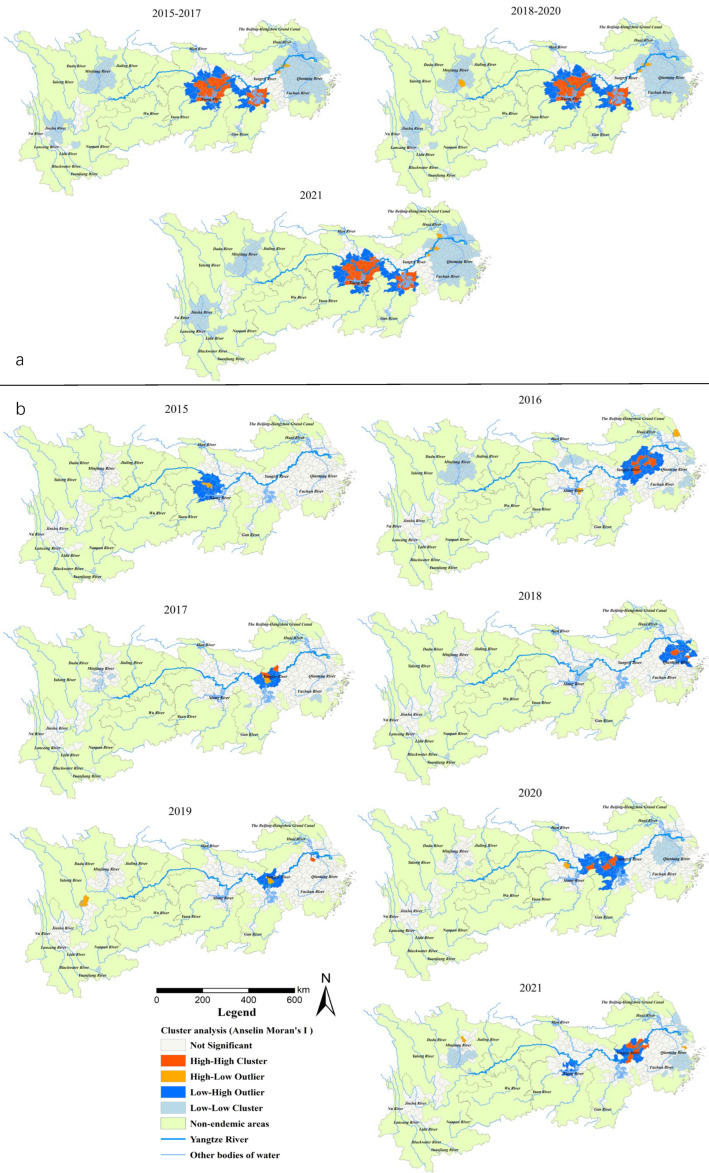
(**a**) Total area of the snail habitats; (**b**) emerging snail habitats. Clusters and outlier areas of snail habitats in the endemic areas in the YEB, 2015–2021.

The Anselin local Moran’s I showed that H-H clusters of all snail habitats were present in 38–39 counties (cities, districts) in alluvial plains, such as in Jianghan Plain (south-central of Hubei Province), Dongting Lake Plain (north-east of Hunan Province) and Poyang Lake Plain (north Jiangxi Province and south-west border of Anhui Province) in the Yangtze River system (Figure 3a), and were relatively stable during 2015–2021. The L-L clusters were present in around 210 counties (southeast of Anhui Province, south Jiangsu Province, north Zhejiang Province and most parts of Shanghai) in the middle and lower reaches of the YEB. For the area of emerging snail habitats, the number of H-H clusters was relatively large, with 11 districts or counties in south Anhui Province (Wuhu, Nanling, Wuwei, Shitai, Zongyang counties and Guichi, Xuanzhou, Daguan, Jiujiang, Huashan, Yushan districts) in 2016, 5 at the south-east border of Hubei Province (Qichun, Yangxin, Jiayu, Wuxue and Chibi) in 2020, and 8 in the southwest of Anhui Province (Nanling, Zongyang, Dongzhi, Guichi, Yian, Jiao districts, Tongcheng and Wuwei) in 2021 (Figure 3b).

**Figure 4:**
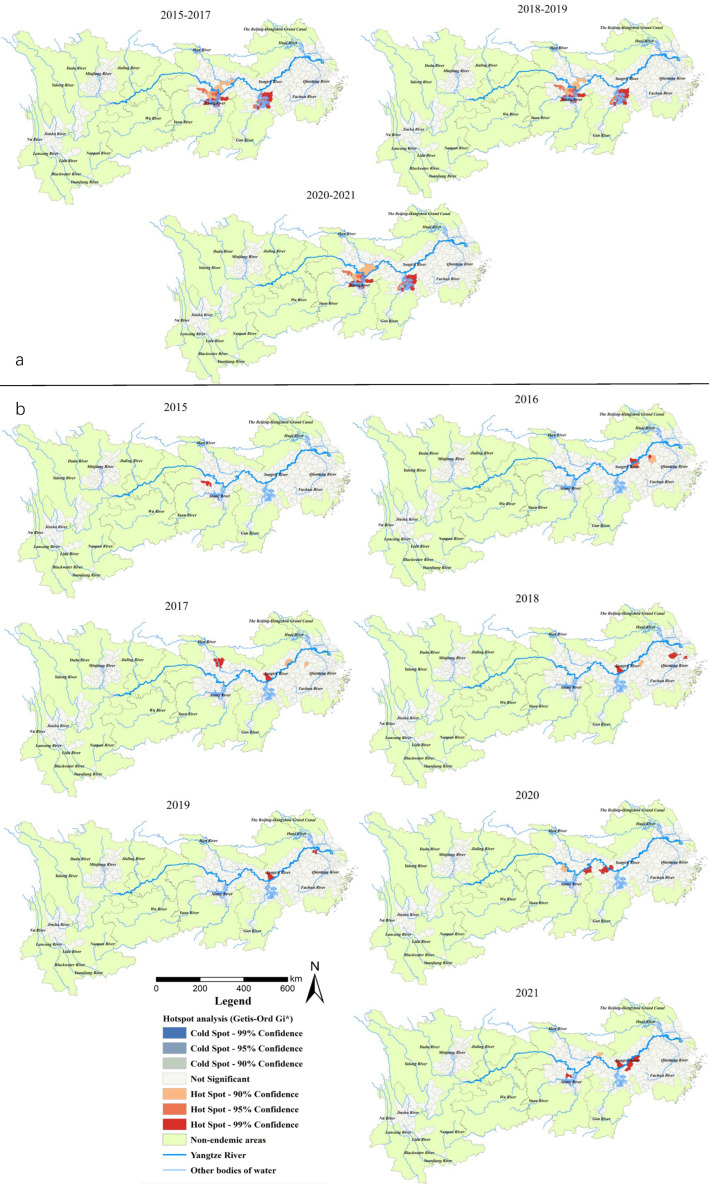
(**a**) Total area of the snail habitats; (**b**) emerging snail habitats. Hotspot areas of snail habitats in the endemic areas in the YEB, 2015–2021.

Gi* statistic hotspot analysis on all snail habitats revealed that there was a subtle difference among the statistically significant hotspots in 2015–2017, 2018–2019 and 2020–2021. In particular, the Caidian District (Hubei Province) became a non-hotspot in 2018, while Honghu city (Hubei Province) became a hotspot in 2020. The hotspots in the three periods mentioned above were generally distributed in 17 counties (cities, districts) in Hubei (Xiantao, Jianli and Shishou cities), Hunan (Junshan District, Yuanjiang city, Li County, Nan County, Anxiang, Huarong, Yueyang, Hanshou and Xiangyin counties) and Jiangxi provinces (Duchang, Poyang, Yugan, Xinjian, Nanchang counties) (Figure 4a). For the area of emerging snail habitats, six hotspots were detected in 2016 (including Wuhu and Zongyang counties, Guichi, Daguan, Xuanzhou districts in Anhui Province and Yushan county in Jiangxi Province) and six in 2021 (Jinghu and Guichi districts, Dongzhi and Susong counties in Anhui Province, Nan County in Hunan Province, Xinzhou District in Hubei Province). Five hotspots were found in 2018 (three in the south of Anhui Provinces, two in Jiangsu Province, and one in Shanghai City) and 2020 (all at the south border of Hubei Province). Furthermore, one, four and two hotspots were detected in 2015, 2017, 2019, respectively, located in the middle and lower reaches along the Yangtze River (Figure 4b).

### 3.4. Spatiotemporal Clusters

**Figure 5:**
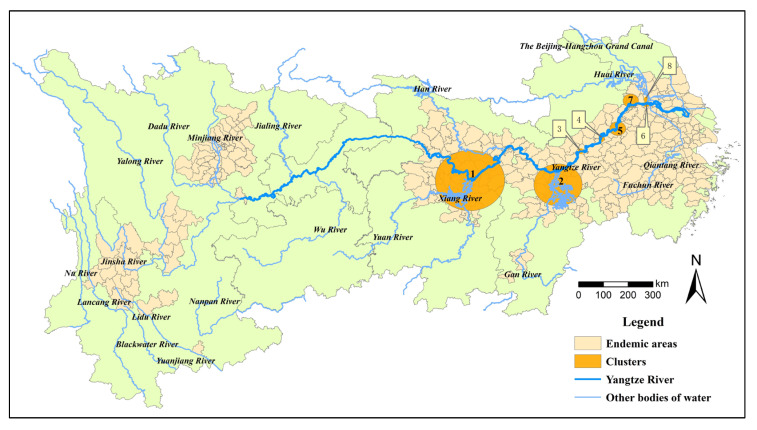
Spatiotemporal cluster areas of snail habitats in the endemic areas in the YEB, 2015–2021.

A total of eight clusters of total snail habitats were detected in endemic areas in the YEB from 2015 to 2021 (Table 2, Figure 5). The most likely cluster was found in 2015–2017, with a radius of 122.21 km, covering 26 counties (cities, districts) in Hubei and Hunan provinces, with the strongest aggregation (LLR = 783,294.08, *p* < 0.01). The second most likely cluster was detected in 2019–2021, covering 18 counties (cities, districts) in Jiangxi Province (LLR = 197,892.11, *p* < 0.001). In addition, another two clusters were investigated in 2015–2017 concentrated in the Daguan district of Anhui Province (LLR = 18,123.69, *p* < 0.01) and the Yian District of Anhui Province (LLR = 5168.83, *p* < 0.01). The remaining cluster areas were successively distributed in Anhui (one cluster) and Jiangsu provinces (three clusters). However, no spatial and temporal aggregation area was found for the emerging snail habitats.

## 4. Discussion

Surveys of snails are an important component of the current schistosomiasis control and elimination program in P.R. of China. A timely and accurate understanding of the spatial and temporal distribution and diffusion trends of snails based on the analysis of snail survey data can provide scientific reference for formulating more accurate interventions to eliminate schistosomiasis or prevent the rebound of schistosomiasis, particularly in regions featured by a very low infection rate in humans, animal hosts and snails [28]. Our study showed that the YEB carries a very high snail burden, with snails distributed in 369,244.05 hm^2^ of settings, accounting for 99.99% of the total snail habitats in China in 2021 [29]. In addition, both the total snail-infested area and the area of emerging snail habitats presented an increasing trend in the YEB from 2015 to 2021. Possible reasons behind this might include the floods that occurred in the lake regions along the Yangtze River in 2016 and 2020 and the difficulty of implementing molluscicides in large areas along the beaches and major lakes of the Yangtze River due to the Yangtze River Protection Law [30].

For the distribution of snails in the YEB from 2015 to 2021, spatial aggregation was detected by the global spatial autocorrelation analysis, which concentrated along the main section and tributaries of the Yangtze River, with obvious water proximity [31,32]. The hotspots identified by local Moran’ I and Getis-Ord Gi* statistics in the YEB were basically consistent. The snail habitats mainly clustered in the middle and lower reaches of the Yangtze River in a stable manner as regards numbers and location of the hotspots during the 7 years. The main breeding environments of the snails were grassland, the lakeshore, river beaches, etc., which were characterized by uncontrollable water levels and a complex vegetation, providing a favorable ecosystem for the survival and reproduction of the snails [33,34]. Mating, spawning, hatching larval growth, migration and diffusion of the snails are carried out along the hydrographic net. Once miracidia of *S. japonicum* penetrate snails, they will proliferate and produce a large number of infectious larvae of *S. japonicum* [35,36,37]. Snails in these areas are difficult to be eliminated completely, and extra applicable integrated methods are needed in addition to molluscicides. In the YEB, the hilly endemic areas in the upper reaches and the broad alluvial plain in the estuary of the Yangtze River were identified as cold spots. The snails are mainly scattered in paddy fields, ponds or irrigation ditches in mountainous and hilly regions and concentrate in riverbanks and irrigation channels in waterway networks in the lower reach of the Yangtze River [38,39].

The spatial aggregation of emerging snail habitats, corresponding to the area of emerging snail habitats, was presented for 2016, 2020 and 2021 and is consistent with the impact of flood disasters on snail diffusion [40]. The hotspots of emerging snail habitats were mainly located in the marshland and hilly endemic areas in the middle and lower reaches of the YEB, and the number of hotspots was relatively large in 2016, 2020 and 2021. The floodings in lake regions and of mountain torrents in hilly regions that occurred in 2016 and 2020 may have largely contributed to the spread of snails [41,42]. As the impacts of floods on snail spreading is delayed according to previous studies [35], further surveillance on snails should be strengthened, particularly in those environments adjacent to snail habitats and greatly affected by flooding in the YEB in the following several years. In addition, emerging snail habitats appeared frequently in recent years due to the construction of wetland parks or seedling production through transplanting trees or reeds containing snails from endemic areas to non-endemic areas [43,44,45,46]. With the call of “To set up the conservation of the Yangtze River and stop its over development”, the ecological restoration will reduce the incidence of floodings and decrease the possibility of snail spreading [47]. However, a large number of economic forests and shelterbelts in the process of ecological restoration will benefit from the development of suitable settings for snail breeding [48]. In addition, social and economic activities such as water conservancy projects, environmental protection projects (removing embankments to discharge floodwater, restoring lakes from eliminating cultivated farmland, wetland construction or protection, land use type change), seedling transplanting, and aquaculture may not only increase the risk of snail diffusion but also form a new suitable environment for snail breeding [37,49,50,51]. We should pay more attention to the human activities impacting on the transmission of schistosomiasis and snail distribution along the YEB.

Comparing the clusters presented by Kulldorff’s space–time scan statistic with the results of hotspot analysis, we found that the distribution of the hotspots was similar to that of the long-duration cluster circles in the space–time scan, indicating that the distribution of the snails in endemic areas in the YEB may not be random. The primary and secondary spatial and temporal clusters were concentrated in the meadow of a lake along the middle reaches, including a total of 44 counties (cities, districts), and 8 (Duchang, Yongxiu, Poyang, Pengze, Yugan, Nanchang counties and Lushan, Gongqing cities in Jiangxi Province) of them were still at the transmission control stage. In addition, the left clusters in the lower reaches included Daguan District and Dongzhi County in Anhui Province, which were also at the stage of transmission control. Due to the slow declining trend in areas of snail habitats and the great difficulties in the eradication of snails in these counties, where snails were mainly distributed on river or lake beaches, the main targets of countermeasures should be to reduce the density of snails and prevent snail spreading from embankments to production areas or living areas, that people or livestock often visit, by hardening the snail habitats, improving the watershed management and designing land consolidation projects, in combination with efficient snail control [52,53]. For complex environments such as hills and mountains, attention should also be paid to the sequential implementation of snail control (e.g., from the surroundings to the center, from high altitude to low altitude in the dam area). Moreover, surveillance should be strengthened and maintained in areas where snails have been eliminated, with no clusters detected in the upper and lower reaches, especially after the occurrence of torrential floods or earthquakes.

It is worth noticing that the distribution of snails is affected by biological, natural, and social-economic factors. With the development of the YEB, the impacts of human activities and other social factors on the distribution of snails has been increasing. In addition, the pressure of environmental protection has hindered snail control by chemicals in areas with aquaculture, wetland construction and protection projects, which might lead to the rebound of schistosomiasis due to the high density of snails and the large area of snail habitats. Thus, we should accelerate the development of eco-friendly molluscicide drugs and implement alternative ecological methods to compress the areas of snail habitats or with high snail density and reduce the transmission risk of schistosomiasis [54].

There are several limitations in our study. One is that we conducted a large-scale analysis on the spatiotemporal patterns of snail distribution based on county-level data, which may hinder some information that can guide snail control precisely. In addition, we only analyzed and discussed the possible factors impacting the snail burden qualitatively based on experts’ experience and knowledge. Based on our findings, further studies could be conducted in a smaller scale to identify key settings or surroundings focusing on the hotspots identified by our study and to assess the influencing factors affecting the distribution of snails or schistosomiasis quantitatively, thus providing scientific information to allocate health resource reasonably and implement interventions precisely to protect public health security in the YEB.

## 5. Conclusions

The YEB carries a massive snail burden that shows a slightly increasing trend. The distribution of snails appeared in a spatial aggregation pattern and mainly concentrated in the middle and lower reaches of the YEB. Therefore, surveillance and interventions against snails on these snail gathering areas and key snail habitats should be strengthened to provide scientific support guiding interventions against schistosomiasis and accelerate the progress of schistosomiasis elimination in China.

## Figures and Tables

**Table 1:** Global spatial autocorrelation analysis of the distribution of the snail habitats in the endemic areas in the YEB from 2015 to 2021.

Year	Total Snail Habitats				Emerging Snail Habitats			
	Moran’s I	Z-Score	*p*-Value	Distribution Model	Moran’s I	Z-Score	*p*-Value	Distribution Model
2015	0.49	15.94	0.00	Clustered	−0.00	−0.34	0.74	Non-clustered
2016	0.49	16.03	0.00	Clustered	0.06	2.26	0.02	Clustered
2017	0.49	15.98	0.00	Clustered	0.01	0.96	0.34	Non-clustered
2018	0.49	15.96	0.00	Clustered	0.12	4.55	0.00	Clustered
2019	0.50	16.13	0.00	Clustered	−0.00	−0.29	0.77	Non-clustered
2020	0.50	15.89	0.00	Clustered	0.18	7.50	0.00	Clustered
2021	0.50	15.95	0.00	Clustered	0.02	0.89	0.37	Non-clustered

**Table 2:** Spatiotemporal cluster analysis on the distribution of actual snail habitats in the endemic areas in the YEB, 2015–2021.

Cluster Area	Cluster Year	Radius (km)	Cluster Center	No. Counties	LLR	RR	*p*-Value
1	2015–2017	122.21	Junshan District	26	783,294.08	9.52	0.00
2	2019–2021	85.20	Duchang County	18	197,892.11	5.35	0.00
3	2015–2017	0.00	Daguan District	1	18,123.69	18.54	0.00
4	2015–2017	0.00	Yi’an District	1	5168.83	4.38	0.00
5	2016–2018	27.81	Jinghu District	5	2294.95	1.95	0.00
6	2016	0.00	Runzhou District	1	155.36	3.55	0.00
7	2021	26.81	Liuhe District	2	84.25	1.38	0.00
8	2017–2018	0.00	Guangling District	1	38.45	1.49	0.00

## Data Availability

Data supporting the conclusions are included within the article. The raw datasets are available from the corresponding author on reasonable request.
